# Metabolic prediction of important agronomic traits in hybrid rice (*Oryza sativa* L.)

**DOI:** 10.1038/srep21732

**Published:** 2016-02-24

**Authors:** Zhiwu Dan, Jun Hu, Wei Zhou, Guoxin Yao, Renshan Zhu, Yingguo Zhu, Wenchao Huang

**Affiliations:** 1 State Key Laboratory of Hybrid Rice, College of Life Sciences, Wuhan University, Wuhan 430072, China; 2 Engineering Research Center for Plant Biotechnology and Germplasm Utilization, Ministry of Education, Wuhan University, Wuhan 430072, China

## Abstract

Hybrid crops have contributed greatly to improvements in global food and fodder production over the past several decades. Nevertheless, the growing population and changing climate have produced food crises and energy shortages. Breeding new elite hybrid varieties is currently an urgent task, but present breeding procedures are time-consuming and labour-intensive. In this study, parental metabolic information was utilized to predict three polygenic traits in hybrid rice. A complete diallel cross population consisting of eighteen rice inbred lines was constructed, and the hybrids’ plant height, heading date and grain yield per plant were predicted using 525 metabolites. Metabolic prediction models were built using the partial least square regression method, with predictive abilities ranging from 0.858 to 0.977 for the hybrid phenotypes, relative heterosis, and specific combining ability. Only slight changes in predictive ability were observed between hybrid populations, and nearly no changes were detected between reciprocal hybrids. The outcomes of prediction of the three highly polygenic traits demonstrated that metabolic prediction was an accurate (high predictive abilities) and efficient (unaffected by population genetic structures) strategy for screening promising superior hybrid rice. Exploitation of this pre-hybridization strategy may contribute to rice production improvement and accelerate breeding programs.

The continuously growing global population and changing climate have pushed crop breeders to find effective breeding strategies for meeting food and energy demands. Although hybrid crops have made tremendous contributions to yield improvements over the past several decades, breeding new elite hybrid combinations is urgently required to guarantee food security. Quantitative hybridization trials and hybrid performance evaluation, which are time-consuming and laborious, form the foundation of present hybrid breeding programs. Breeding procedures are even tougher for self-pollinated plants such as rice and wheat compared with cross-pollinated plants.

Prediction of hybrid performance based on parental information appears to be a practicable method to increase breeding efficiency. However, parental phenotypes have exhibited low accuracy for the prediction of hybrid performance^1^,^2^. Additionally, isozymes have proven to be unreliable for the prediction of F_1_ yields and heterosis^3^. Then, parental genetic distances based on AFLP (Predictive abilities: 0–0.97, 0.161–0.699 and 0.06–0.8, respectively)^4^,^5^,^6^, InDel (0.049–0.21)^7^, RAPD (0.069–0.785)^8^, RFLP (−0.028–0.773)^9^, SNP (0.26–0.56 and 0.3–0.46)^1^,^10^, and SSR (0.06–0.82, 0.069–0.785, and −0.028–0.773, respectively)^6^,^8^,^9^ DNA markers have been widely used to predict hybrid phenotypes, heterosis or combing ability in maize (*Zea Mays* L.)^5^,^6^,^10^, rice (*Oryza Sativa* L.)^7^,^8^,^9^, sunflowers (*Helianthus annuus* L.)^4^, and *Arabidopsis thaliana*^1^. Some prediction methods based on DNA markers produced no significant correlation or low predictive ability between parental genetic distance and hybrid performance^9^,^10^. Meanwhile, DNA markers were found to be appropriate for the prediction of specific traits^5^,^6^,^9^ and had high predictive ability for populations consisting of genetically related parental lines^4^,^8^. Additionally, parental transcriptome-based predictions of hybrids showed high predictive ability for intra-pool crosses but not inter-pool crosses^11^. Generally speaking, although various types of biomarkers, populations and mathematical models have been tested in various species, problems of low predictive ability or limited application ranges of the prediction approaches remain. And worldwide, numerous studies of hybridization are still performed every year.

The metabolome has manifested potential roles in facilitating crop breeding strategies over the past decades^12^,^13^. The results from a tomato IL population showed that 73% of the total analysed metabolites were significantly associated with the whole-plant phenotypes^14^. *Arabidopsis thaliana* biomass could be described as a function of metabolic compositions with high predictive power^15^,^16^,^17^. Moreover, metabolite profiling of a rice recombinant inbred line population and diverse accessions showed promise in bridging the gap between the genome and phenome^18^,^19^. Importantly, parental metabolites as biomarkers have been tested for the prediction of traits such as yield in maize^20^, biomass and tolerance in *Arabidopsis thaliana*^21^,^22^ and postharvest quality traits in potatoes (*Solanum tuberosum* L.)^23^.

In the present study, a complete diallel cross population was built with eighteen rice inbred lines as the parents. Three polygenic traits—yield per plant, heading date and maturation stage plant height—were predicted in the hybrids using 525 metabolites with the partial least square regression method. The predictive abilities ranged from 0.858 to 0.977 for the three traits. The population structure and cytoplasmic effects had slight influences on the predictive ability.

## Results

### Population structure and hybrid performance

**Figure 1:**
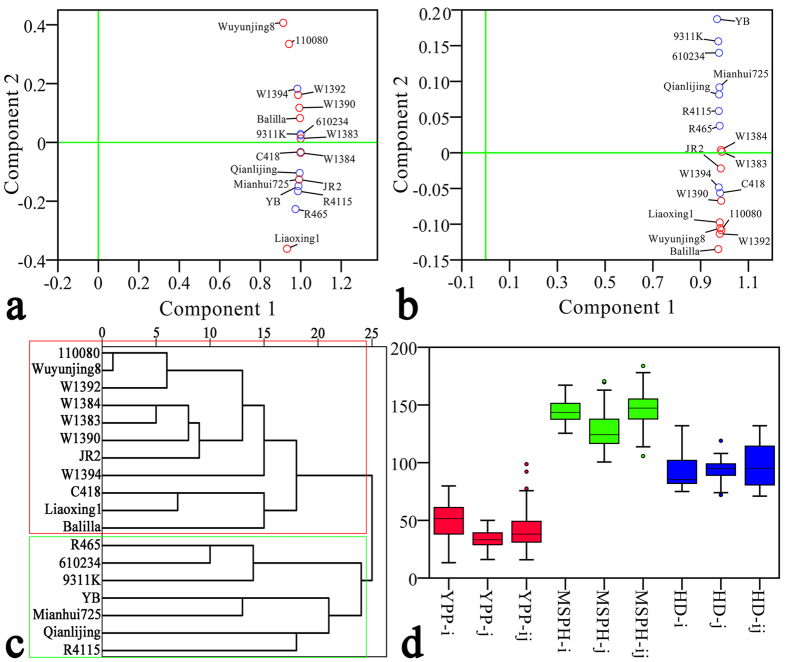
Population structure and hybrid phenotypes in three sub-groups. (**a**) Principal component analysis (PCA) of the eighteen rice inbred lines with data for three agronomic traits. Three *indica* varieties were grouped into the *japonica* group, and three *japonica* varieties were grouped into the *indica* group. Red circles indicate *japonica* varieties, and blue circles indicate *indica* varieties. Solid green lines represent the zero values for each component. (**b**) PCA of the eighteen inbred lines with metabolite profiling data. All 525 analytes were used in the PCA. Two *indica* varieties were grouped into the *japonica* group. (**c**) Dendrogram of the eighteen inbred lines. All eighteen inbred lines were divided into two clear groups. Consistent with the grouping result in (**b**), only two *indica* varieties were in the *japonica* group. The red solid box indicates the *japonica* group, and the green solid box indicates the *indica* group. (**d**) Hybrid phenotypes in three subgroups. The yield per plant (YPP), maturation stage plant height (MSPH) and heading date (HD) were evaluated.

Eighteen rice inbred lines were chosen as parental lines for the complete diallel cross design (see Supplementary Table S1). All the parental lines were divided into equal *indica* (F_i_ values from 0.39 to 1) and *japonica* (F_i_ values from 0 to 0.33) groups based on the InDel marker estimating method (see Supplementary Table S1)^24^. Principal component analysis (PCA) was also applied to the parental phenotypic and metabolic data to discriminate between the parental lines. PCA of the parental phenotypic performance showed that three *indica* varieties were divided into the *japonica* group and three *japonica* varieties were in the *indica* group (Fig. 1a). At the metabolic level, a total of 525 analytes were detected in all eighteen inbred lines. Unexpectedly, PCA for the parental metabolic parameters produced a similar grouping result as the consequences of the InDel markers and was also highly similar to the dendrogram (Fig. 1b,c).

Three important agronomic traits were measured: yield per plant (YPP), maturation stage plant height (MSPH), and heading date (HD). Prior to the analysis, the hybrids were divided into an *indica*-*indica* group (i-group), a *japonica*-*japonica* group (j-group) and an *indica*-*japonica* group (ij-group) based on the group to which the corresponding parental lines belonged. For the hybrid phenotypes (trait values *per se*), the highest mean YPP was in the i-group, the lowest was in the j-group, and the ij-group was between the i- and j-group (Fig. 1d). For MSPH and HD, the highest means were observed for the ij-group, which was consistent with the phenomenon that hybridization between inter-subspecific rice has stronger biomass heterosis (increased plant height) and a later heading date. Box plots depicting the relative low-parent heterosis (LPH), mid-parent heterosis (MPH), better-parent heterosis (BPH) and specific combining ability (SCA) for the three traits are shown in Supplementary Fig. S1a,b. For YPP, the hybrid trait values *per se* had the closest relationship with SCA (see Supplementary Table S2). Trait values of MSPH *per se* had the closest relationship with BPH, and HD was most closely associated with MPH.

### Predictions of hybrid performance based on parental traits and genetic distance

First, the sum trait values of the parents (sum), the differences between the parents (difference) and the ratios of the parents (ratio) were used as predictive variables. The highest correlation coefficient (0.714) was obtained between the HD and the sum values of the parents (see Supplementary Table S3). After performing linear regression analysis of the sum HD values of the parents and the hybrid HD values, the predicted HD values and measured true values displayed dramatic deviances (see Supplementary Fig. S2). Therefore, the parental traits were not appropriate for the prediction of hybrid performance.

Second, to evaluate whether genetic distance based on parental metabolic data was suitable for predicting hybrid performance, squared Euclidean distances based on all 525 analytes were calculated to analyse the correlations (see Supplementary Table S4). The Pearson correlation coefficients between genetic distance and hybrid performance shown in Supplementary Table S5 suggested that, although genetic distance was significantly correlated with some traits (such as YPP and MSPH), the predictive abilities were quite low, and some traits were not closely correlated with genetic distance.

### Stepwise linear regression and partial least square (PLS) regression with metabolic data and hybrid performance

Next, the sums, differences and ratios of the parents’ relative metabolite levels were calculated, and stepwise linear regression was used to identify the appropriate metabolic predictors. The adjusted R^2^ values in Supplementary Table S6 shows that the highest value was 0.677 for HD, while no metabolite could be used for the regression of traits such as SCA-YPP. Furthermore, the predictive abilities of different traits varied widely, and they were not sufficiently high for most traits.

**Figure 2:**
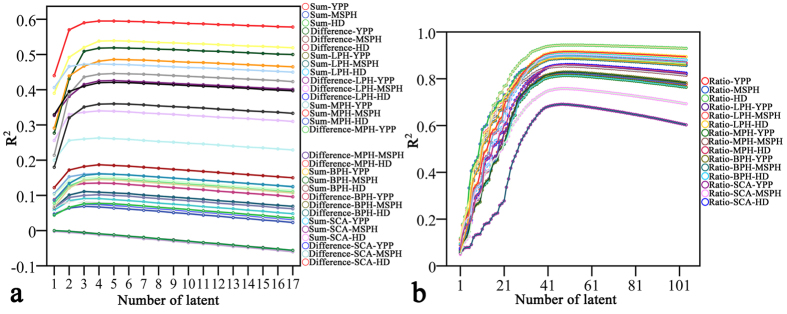
Relationships between PLS latent numbers and R^2^ values. (**a**) Sums and differences between parental relative metabolite levels were used as predictive variables for the hybrid phenotypes, relative heterosis and specific combining ability in PLS regression. The R^2^ values varied largely between different traits. (**b**) Ratios of parental relative metabolite levels were used for hybrid performance prediction. R^2^ values of most traits were highest (above 0.8) at approximately 50 latents. YPP = Yield per plant, MSPH = Maturation stage plant height, HD = Heading date, LPH = Low-parent heterosis, MPH = Mid-parent heterosis, BPH = better-parent heterosis, SCA = Specific combining ability.

Then, partial least square regression was used to create a prediction model with metabolic data of the three traits. In Fig. 2a, 17 latent factors were extracted for all the traits using the sums and differences of the parents’ relative metabolite levels as variables. Unfortunately, the highest R^2^ value obtained was 0.6, and some hybrid traits could not be predicted using these parental variables.

Surprisingly, however, high R^2^ values were obtained when the ratios of parental metabolic data were used as predictive variables (Fig. 2b). For this analysis, 104 to 107 latent factors were extracted from the metabolic data; most of the traits exhibited the highest R^2^ values at approximately 50 latent factors. Then, the number of latent factors at the top R^2^ value for each trait was fixed to obtain the values of variable importance in the projection (VIP) of each variable. Because Latent Factor 1 explained the largest proportion of the variance, the VIP values of Latent Factor 1 were reordered to reduce the number of variables. After excluding variables whose VIP values were smaller than 1, the remaining variables were used for PLS regression. Finally, although more than 300 variables were excluded from the regression model, and only slight decreases were observed in R^2^ values compared with the values obtained using all 525 metabolites (Table 1). Among the 15 predicted traits, LPH-YPP used the fewest (149) variables and LPH-HD used the most variables (196).

Considering that 149–196 is still a quite large number of variables and might result in overfitting^22^, the VIP values of each variable were reordered again in the PLS regression model to minimize the amount of variables. Unfortunately, the R^2^ values decreased dramatically.

### Metabolic prediction of agronomic traits by PLS regression

**Figure 3:**
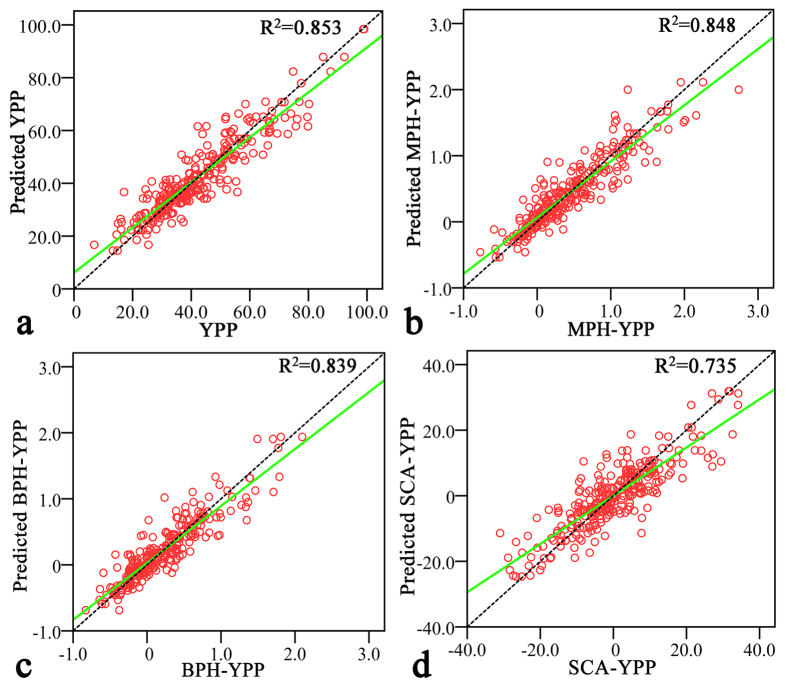
Observed and predicted values of YPP, MPH-YPP, BPH-YPP, and SCA-YPP. (**a**) Relationships between observed yield per plant and predicted yield per plant. Predicted values were calculated with the equations based on variable coefficients in the PLS regression results. The horizontal axis represents the observed values, and the vertical axis represents the predicted values. The green solid line represents the total fit line, and the black dotted line is *y* = *x*. (**b**–**d**) Relationships between the observed relative mid-parent heterosis, better-parent heterosis, specific combining ability of YPP and the corresponding predicted values. YPP = Yield per plant, LPH = Low-parent heterosis, MPH = Mid-parent heterosis, BPH = better-parent heterosis, SCA = Specific combining ability.

The coefficient of each variable in the PLS regression results was used to build the equations (see Supplementary Table S7). Then, a predicted value was assigned for every trait in each hybrid. The relationship between the observed YPP (Fig. 3a), MPH-YPP (Fig. 3b), BPH-YPP (Fig. 3c), and SCA-YPP (Fig. 3d) values and the predicted values demonstrated the high accuracy for this metabolite-based prediction method. The lowest predictive ability was 0.858 with SCA-YPP and the highest was 0.924 with YPP. High predictive abilities were also achieved through the PLS regression approach for the remaining traits (Table 2).

**Figure 4:**
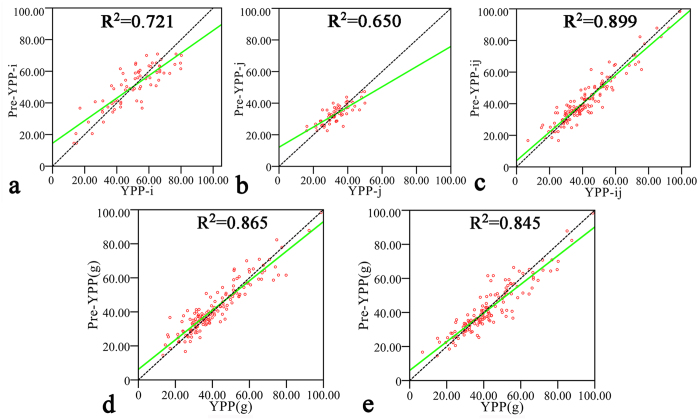
Observed and predicted YPP values in three subgroups and two reciprocal groups. (**a**–**c**) Relationships between observed yield per plant and predicted values in the i, j and ij three subgroups. The entire hybrid population was divided into three subgroups based on whether a parental line was *indica* or *japonica* (Fig. 1b). The predictive abilities in different population structures showed only slight changes. (**d**–**e**) Relationships between observed yield per plant and predicted yield per plant in reciprocal hybrids. The whole population was divided into two groups of reciprocal hybrids to test cytoplasmic effects on predictive ability. The results demonstrated that different cytoplasms only weakly influenced predictive ability. YPP = Yield per plant.

Because the complete diallel cross population consisted of rice inbred lines ranging from typical *indica* to typical *japonica*, the predictive abilities might vary with different hybridization groups. Thus, the predictive abilities for the i-group, j-group and ij-group were calculated to validate the stability of the prediction model (Table 2). As shown in Fig. 4a,b, the predictive abilities of YPP in the i-group and j-group were 0.849 and 0.806, respectively. Surprisingly, the predictive ability of the ij-group was as high as 0.948 (Fig. 4c). Moreover, the predictive abilities of relative heterosis and SCA of YPP were all the highest for the ij-group, thereby showing a promising approach for exploiting inter-subspecific heterosis^25^.

Finally, the cytoplasm from the reciprocal parental lines might have different influences on hybrid performance; these differences might be obstacles to applying the metabolic prediction strategy in screening for potential sterile and restorer lines. Hence, the whole population was divided into two sets of reciprocal hybrids to test whether cytoplasmic effects affected the predictive ability. The results demonstrated that different cytoplasms had weak influences on the predictive ability (see Fig. 4d,e and Supplementary Table S8).

## Discussion

Accuracy and efficiency are two pivotal indicators for the application of a biotechnology-assisted prediction model in breeding. Because predictive ability might be heavily influenced in populations consisting of genetically distant lines^4^,^11^; therefore, assessing whether a prediction model is appropriate is largely determined by its predictive abilities in different genetic structures. To avoid incomplete bias prediction, the predictive model should be built on representative inbred lines. In addition, the richer the information on these inbred lines, the higher the potential accuracy of the model. In this experiment, eighteen rice inbred lines were used to build a prediction model. These lines were collected from a wide range of locations (China, Italy and India) and possessed different degrees of *indica* or *japonica* contents and variable general combining abilities (see Supplementary Table S1). To some extent, they represented a large range of rice accessions that were suitable for building the prediction model. Therefore, this model may also be applicable to rice inbred lines that were not included in this experiment.

The relationship between parental metabolite profiling and hybrid performance should be nonlinear^26^ and was treated as a megavariate quantitative structure-activity relationship in this study^27^. Because of its powerful multi-dimensional information regression ability, partial least squares projections to latent structures^28^ were applied to build the hybrid trait prediction model. Meanwhile, because cross-validation is built into the PLS algorithms^27^, the predictive significance is assessed and predictive abilities can be insured for every trait. In addition, taking advantage of the values of variable importance in the projection, the low contribution variables were excluded and the representative variables were reserved for the equations^22^. The high predictive abilities (0.858–0.977) in Table 2 demonstrate that PLS matched the metabolic prediction model quite well.

Metabolites have close connections with phenotypes^14^,^17^,^19^. Before determining the exact function of a metabolite, correlation analysis between the relative metabolite levels and phenotypes can be used as a type of rough estimating method^29^. In this study, different groups of metabolites were exploited to predict various traits. Because specific metabolites may have single or multiple functions in different pathways, metabolites such as saccharic acid, p-Cresol, and triacontanoic acid methyl ester were found to be predictive variables for two or more traits (see Supplementary Table S9). Different correlation levels were detected for the metabolites that were predictive variables for all three traits (see Supplementary Table S10). Some of these overlapping metabolites manifested significant positive and negative correlations with different traits, which might be the origin of the balance between component traits^30^ or outcomes of feedback regulation of the biological networks involved in complex traits^31^. Meanwhile, only a single value was used to represent reciprocal hybrids when calculating the ratios of relative parental metabolite levels. Ignorance of the relationship between the female parent and male parent might decrease the predictive abilities for the reciprocal hybrids. However, almost no predictive ability change was found between the reciprocal hybrids. Therefore, close balances might exist between metabolites and their associations might be controlled by the rules of chemistry^26^. The influences of the complex associations between metabolites on the hybrid trait prediction model remain unknown.

Nevertheless, predictable metabolites in the prediction model might simply have high predictive abilities for the traits. Therefore, biological functions of the predictive variables in this study were analysed to verify their contributions to the prediction model. Fructose (a predictive variable for yield per plant and maturation stage plant height) contributes strongly to the metabolic efficiency of rosette fresh weight and protein concentration in *Arabidopsis*^29^. Ferulic acid, a phenolamide^32^, plays an important role in plant development^33^ and is a predictive variable for plant height. Spermidine (a predictive variable for heading date and yield per plant) is associated with floral induction and development^33^,^34^. Furthermore, significant correlations have been found between alanine and biomass or yield-related traits in *Arabidopsis*^35^ and tomatoes^14^. Hence, our results demonstrate that the high predictive abilities for the three traits were not merely coincidental.

However, the metabolic prediction model might not be directly applicable to other breeding programs. In this study, metabolite profiling of 15-day-old seedlings of the parental lines was chosen to provide the predictors for hybrid traits. Thus, the metabolite profiling data were only a snapshot of the whole plant growth procedure. However, plant metabolism is a highly dynamic system that changes substantially over time^20^,^36^. Therefore, although high predictive abilities for three agronomic traits were obtained in this study, the metabolite profiling data presented here might be unsuitable for traits such as grain number per panicle or tiller number per plant. Therefore, the appropriate time points must be adapted when forecasting other polygenic traits from assembled metabolite profiling data.

Furthermore, the performance of a plant in nature is the consequence of the combination of genetic information and environmental influences; thus, the effects of environmental inputs on metabolome-assisted breeding strategies might need to be taken into account^26^. Metabolite profiling data of naturally growing parental seedlings in a specific field environment should be more accurate for the prediction of hybrid performances in corresponding locations.

In summary, a reliable and efficient metabolic prediction strategy was provided by combining parental metabolite profiling with a PLS regression method for rice hybrids. The high predictive abilities for three agronomic traits were implemented with respect to hybrid phenotypes, relative heterosis and specific combining ability. The predictive abilities for the three traits were only slightly influenced by population structures (genetic relatedness) or cytoplasmic effects. Additionally, metabolite-based prediction might be more suitable for traits such as resistance (susceptibility) or tolerance ability^21^,^23^,^37^ because small molecules function more directly in these defence processes by confronting biotic/abiotic pressures.

## Methods

### Plant materials

The eighteen rice inbred lines were selected based on their proportions of *indica*-*japonica* content using the InDel marker estimation method^24^. A complete diallel cross design was used. All the hybrid seeds were produced through manual emasculation at the Hybrid Rice Hainan Experimental Base of Wuhan University in Lingshui (N18° 30′ 22.12″, E110° 2′ 10.72″), Hainan Province, China, in 2012. Seedlings of the eighteen inbred lines and 306 hybrids were planted with a randomized block design in three replicates at the Hybrid Rice Ezhou Experimental Base of Wuhan University in Ezhou (N30° 22′ 19.82″, E114° 44′ 59.17″), Hubei Province, China, in June 2012. Ten plants were planted per row with a spacing of 16.5 × 26.4 cm. Four cytoplasmic male-sterile plants (named YTA) were planted around these experimental plants to decrease marginal effects. The middle five plants of each replicate were chosen for data collection. Three agronomic traits were evaluated: grain yield per plant, heading date and maturation stage plant height. Means among the replications were calculated for each trait and used in the data analysis.

For metabolite profiling analysis, seeds of the eighteen rice inbred lines were first submerged in water for two days at 28 °C. Then, the seeds were placed in an incubator at 28 °C for 24 h to accelerate germination. Next, seedlings at approximately the same stage were transferred to soil containers. Three random replicates were applied for each inbred line; the spacing between seedlings was 2 × 2 cm. Finally, all the seedlings were placed in a phytotron with a temperature of 28 °C, 70% relative humidity and an 8 h light/16 h dark photoperiod. On day 15, 100 mg of the seedlings (root excluded) were collected into 2 ml EP tubes for each replicate and immediately frozen in liquid nitrogen.

### Gas chromatography mass spectrometry–based metabolite profiling

For each sample, 0.4 ml of methanol-chloroform (V_methanol_:V_chloroform_ = 3:1) and 20 μl of ribitol (0.2 mg/ml stock in dH_2_O, Sigma-Aldrich Co. LLC., USA) were added as internal standards. After vortex mixing for 10 s, steel balls were placed into EP tubes and the samples were homogenized with a ball mill (JXFSTPRP-24, Shanghai Jingxin Experimental Technology, Shanghai, China) for 5 min at 55 Hz. Then, the samples were centrifuged for 15 min at 12,000 rpm at 4 °C. The supernatant (approximately 0.4 ml) was transferred to a new 2 ml GC/MS glass vial. An equal volume of approximately 13 μl (based on the number of samples) from each sample was transferred into a new 2 ml GC/MS glass vial as a mixed sample for quality control. Then, the extracts were dried in a vacuum concentrator without heating at 30 °C for approximately 1.5 h. Next, 80 μl of methoxymethyl amine salt (dissolved in pyridine, final concentration of 20 mg/ml) was added to the dried extracts and incubated at 37 °C for 2 h in an oven after mixing and sealing. The lids were opened, and 100 μl of BSTFA (containing 1% TCMS, v/v, Regis Technologies, Inc., USA) was added to each sample; then, the samples were resealed and incubated at 70 °C for an hour. When the samples had cooled to room temperature, 10 μl of FAMEs (standard mixture of fatty acid methyl esters, 1 mg/ml C8-C16 and 0.5 mg/ml C18-C30 in chloroform) was added to the mixed sample. Then, the sample was mixed well for GC-MS analysis.

GC/TOF MS analysis was performed using an Agilent 7890 (Agilent Technologies, USA) gas chromatograph system coupled with a Pegasus HT time-of-flight mass spectrometer (LECO Corporation, USA). The system utilized a DB-5 MS capillary column coated with 5% diphenyl cross-linked with 95% dimethyl polysiloxane (30 m × 250 μm inner diameter, 0.25 μm film thickness; J&W Scientific, Folsom, CA, USA). A total of 1 μl of the sample was injected in the splitless mode with helium as the carrier gas. The front inlet purge flow was 3 ml min^−1^, and the gas flow rate through the column was 20 ml min^−1^. The initial temperature was held constant at 50 °C for 1 min, followed by a 10 °C per min ramp up to 330 °C, and then maintained for 5 min at 330 °C. The injection, transfer line, and ion source temperatures were 280 °C, 280 °C, and 220 °C, respectively. The energy was −70 eV in electron impact mode. Spectra were recorded in full-scan mode with an m/z range of 85–600, at a rate of 20 spectra per second after a solvent delay of 366 s.

The Chroma TOF 4.3X software from LECO Corporation and the LECO-Fiehn Rtx5 database^38^ were used for raw peak extraction, data baseline filtering and calibration, peak alignment, deconvolution analysis, peak identification and integration of the peak area. The RI (retention time index) method was used for peak identification; the RI tolerance was 5000.

### Data analysis

Relative low-parent heterosis (LPH), mid-parent heterosis (MPH), better-parent heterosis (BPH) and specific combining ability (SCA) for each trait were calculated using the following equations: LPH = (F_1_-P_Low_)/P_Low_, MPH = (F_1_-P_Mean_)/P_Mean_, BPH = (F_1_-P_High_)/P_High_, GCA_i_ (general combining ability) = P_i._-P.., and SCA_ij_ = P_ij_-P..-P_i._-P_j._^39^. For the heading date, earlier heading indicated positive heterosis. F_1_ is the trait value of the hybrid; P_Low_, P_Mean_ and P_High_ are the low value, mean value and high value of the two corresponding parents, respectively. P_ij_ is the trait value of the hybrid from parent i and parent j, P.. is the mean of all the hybrids, P_i._ is the mean of the hybrids from parent i and P_j._ is the mean of the hybrids from parent j. The relative metabolite levels of each analyte were obtained by calculating the ratio between the areas of the analyte and its corresponding ribitol in each repeat. Means among the replications were used as the raw metabolic data. Metabolic data were log_2_-transformed for statistical analysis. Analytes with the same annotations but different mass and similarity (such as 2-hydroxypyridine), the suffixes −1, −2, and −3 were added in retention time order to differentiate between them in the analyses. Microsoft Excel 2010 (Microsoft, USA) was used to calculate the sums (sum = (Female P+Male P)*0.5) of the parents, differences (difference = Female P-Male P) between the parents, and ratios (ratio = Female P/Male P) of the parents’ phenotypic data. Female P represents the mean of the female parent values and male P represents the mean of the male parent values. When parental metabolic data were used as predictive variables, only single sum, difference and ratio values were calculated for the reciprocal hybrids. For example, for reciprocal hybrids *YB/Balilla* and *Balilla/YB*, if the relative level of Analyte X is **a1** for *YB* and **a2** for *Balilla*, the sum, difference, and ratio values for Analyte X of these reciprocal hybrids are (**a1 **+ **a2**)*0.5, **a1 – a2**, and **a1**/**a2**, respectively. The reason for this calculation approach is explained in the Discussion. Correlation analyses and regression analyses were performed with IBM SPSS Statistics 20 (IBM, USA). Average linkage between groups was chosen as the cluster method in the hierarchical cluster analysis. Squared Euclidean distance was calculated as the genetic distance. Principle component analysis was achieved through factor analysis, and eigenvalues greater than 1 were extracted; maximum iterations for convergence was 25; and the non-rotation method was applied for factor analysis. In stepwise linear regression, the probability of F was used as the stepping method criteria. The entry value was 0.05, and the removal value was 0.10. Partial least squares regression was conducted with the PLS extension bundle for SPSS. Main effects were used to specify model effects. Maximum numbers of latent factors were adjusted until no more latent factors could be extracted. Then, in the Proportion of Variance Explained table, the number of latent factor to a trait was determined where the corresponding adjusted R-square value was at the highest. After the number of latent factor was fixed, all the 525 parental metabolic data were applied to PLS again. And variable importance in the projection of Factor 1 was used for reordering. Parameters of independent variables were used to calculate the corresponding values of the dependent variables. Predictive ability was defined based on the Pearson correlation (2-tailed) coefficient of the predicted and observed values^2^,^20^.

## Additional Information

**How to cite this article**: Dan, Z. *et al.* Metabolic prediction of important agronomic traits in hybrid rice (*Oryza sativa* L.). *Sci. Rep.* **6**, 21732; doi: 10.1038/srep21732 (2016).

## Supplementary Material

Supplementary Dataset 1

Supplementary Table S7

Supplementary Information

## Figures and Tables

**Table 1:** R^2^ values of PLS regression with all 525 variables and with variables with VIP values larger than 1.

Traits	Latent number*	R^2^	Variable number	Latent number^†^	R^2^
YPP	52	0.824	184	70	0.807
MSPH	51	0.890	161	77	0.871
HD	57	0.944	193	78	0.938
LPH-YPP	49	0.860	149	66	0.828
LPH-MSPH	52	0.893	181	66	0.884
LPH-HD	54	0.916	196	78	0.905
MPH-YPP	51	0.814	162	68	0.784
MPH-MSPH	49	0.855	181	73	0.838
MPH-HD	53	0.896	182	72	0.884
BPH-YPP	52	0.829	166	69	0.810
BPH-MSPH	52	0.887	164	78	0.869
BPH-HD	55	0.905	178	73	0.896
SCA-YPP	48	0.691	183	64	0.661
SCA-MSPH	50	0.758	159	79	0.704
SCA-HD	53	0.861	171	76	0.844

^*^Latent number for different traits of PLS analysis with all 525 variables. ^†^Latent number for different traits of PLS analysis with variables with VIP values larger than 1. YPP = Yield per plant, MSPH = Maturation stage plant height, HD = Heading date, LPH = Relative low-parent heterosis, MPH = Relative mid-parent heterosis, BPH = Relative better-parent heterosis, SCA = Specific combining ability.

**Table 2:** Predictive abilities of the whole population and three subgroups for the three polygenic traits.

Traits	Whole population	i-group	j-group	ij-group
YPP	0.924	0.849	0.806	0.948
MSPH	0.951	0.911	0.901	0.956
HD	0.977	0.987	0.937	0.977
LPH-YPP	0.931	0.927	0.906	0.940
LPH-MSPH	0.954	0.931	0.897	0.965
LPH-HD	0.964	0.963	0.894	0.968
MPH-YPP	0.921	0.883	0.911	0.940
MPH-MSPH	0.938	0.933	0.856	0.948
MPH-HD	0.954	0.963	0.838	0.957
BPH-YPP	0.916	0.848	0.926	0.935
BPH-MSPH	0.949	0.933	0.904	0.949
BPH-HD	0.931	0.953	0.948	0.913
SCA-YPP	0.858	0.783	0.873	0.898
SCA-MSPH	0.885	0.940	0.736	0.868
SCA-HD	0.940	0.963	0.916	0.927

YPP = Yield per plant, MSPH = Maturation stage plant height, HD = Heading date, LPH = Relative low-parent heterosis, MPH = Relative mid-parent heterosis, BPH = Relative better-parent heterosis, SCA = Specific combining ability.

## References

[b1] Steinfath M. *et al.* Prediction of hybrid biomass in *Arabidopsis thaliana* by selected parental SNP and metabolic markers. Theor Appl Genet 120, 239–247 (2010). 19911163 10.1007/s00122-009-1191-2 PMC2793375

[b2] Feher K. *et al.* Deducing hybrid performance from parental metabolic profiles of young primary roots of maize by using a multivariate diallel approach. PLoS ONE 9, e85435 (2014). 24409329 10.1371/journal.pone.0085435 PMC3883692

[b3] Yu C. Y., Hu S. W., Zhao H. X., Guo A. G. & Sun G. L. Genetic distances revealed by morphological characters, isozymes, proteins and RAPD markers and their relationships with hybrid performance in oilseed rape (*Brassica napus* L.). Theor Appl Genet 110, 511–518 (2005). 15578151 10.1007/s00122-004-1858-7

[b4] Reif J. C., Zhao Y., Würschum T., Gowda M., Hahn V. & Léon J. Genomic prediction of sunflower hybrid performance. Plant Breed 132, 107–114 (2013).

[b5] Schrag T. A. *et al.* Molecular marker-based prediction of hybrid performance in maize using unbalanced data from multiple experiments with factorial crosses. Theor Appl Genet 118, 741–751 (2009). 19048224 10.1007/s00122-008-0934-9

[b6] Schrag T. A. *et al.* Prediction of hybrid performance in maize using molecular markers and joint analyses of hybrids and parental inbreds. Theor Appl Genet 120, 451–461 (2010). 19916002 10.1007/s00122-009-1208-x

[b7] Dan Z. *et al.* Balance between a higher degree of heterosis and increased reproductive isolation: a strategic design for breeding inter-subspecific hybrid rice. PLoS ONE 9, e93122 (2014). 24667442 10.1371/journal.pone.0093122 PMC3965518

[b8] Xiao J., Li J., Yuan L., McCouch S. & Tanksley S. D. Genetic diversity and its relationship to hybrid performance and heterosis in rice as revealed by PCR-based markers. Theor Appl Genet 92, 637–643 (1996). 24166385 10.1007/BF00226083

[b9] Zhang Q. F., Zhou Z. Q., Yang G. P., Xu C. G., Liu K. D. & Saghai Maroof M. A. Molecular marker heterozygosity and hybrid performance in indica and japonica rice. Theor Appl Genet 93, 1218–1224 (1996). 24162533 10.1007/BF00223453

[b10] Windhausen V. S. *et al.* Effectiveness of genomic prediction of maize hybrid performance in different breeding populations and environments. G3: Genes|Genomes|Genetics 2, 1427–1436 (2012). 23173094 10.1534/g3.112.003699 PMC3484673

[b11] Frisch M., Thiemann A., Fu J., Schrag T. A., Scholten S. & Melchinger A. E. Transcriptome-based distance measures for grouping of germplasm and prediction of hybrid performance in maize. Theor Appl Genet 120, 441–450 (2010). 19911157 10.1007/s00122-009-1204-1

[b12] Fernie A. R. & Schauer N. Metabolomics-assisted breeding: a viable option for crop improvement? Trends Genet 25, 39–48 (2009). 19027981 10.1016/j.tig.2008.10.010

[b13] Stitt M., Sulpice R. & Keurentjes J. Metabolic networks: how to identify key components in the regulation of metabolism and growth. Plant Physiol 152, 428–444 (2010). 20018593 10.1104/pp.109.150821 PMC2815907

[b14] Schauer N. *et al.* Comprehensive metabolic profiling and phenotyping of interspecific introgression lines for tomato improvement. Nat Biotechnol 24, 447–454 (2006). 16531992 10.1038/nbt1192

[b15] Meyer R. C. *et al.* The metabolic signature related to high plant growth rate in *Arabidopsis thaliana*. Proc Natl Acad Sci USA 104, 4759–4764 (2007). 17360597 10.1073/pnas.0609709104 PMC1810331

[b16] Lisec J. *et al.* Identification of heterotic metabolite QTL in *Arabidopsis thaliana* RIL and IL populations. Plant J 59, 777–788 (2009). 19453458 10.1111/j.1365-313X.2009.03910.x

[b17] Sulpice R. *et al.* Starch as a major integrator in the regulation of plant growth. Proc Natl Acad Sci USA 106, 10348–10353 (2009). 19506259 10.1073/pnas.0903478106 PMC2693182

[b18] Gong L. *et al.* Genetic analysis of the metabolome exemplified using a rice population. Proc Natl Acad Sci USA 110, 20320–20325 (2013). 24259710 10.1073/pnas.1319681110 PMC3864304

[b19] Chen W. *et al.* Genome-wide association analyses provide genetic and biochemical insights into natural variation in rice metabolism. Nat Genet 46, 714–721 (2014). 24908251 10.1038/ng.3007

[b20] Riedelsheimer C. *et al.* Genomic and metabolic prediction of complex heterotic traits in hybrid maize. Nat Genet 44, 217–220 (2012). 22246502 10.1038/ng.1033

[b21] Korn M., Gartner T., Erban A., Kopka J., Selbig J. & Hincha D. K. Predicting *Arabidopsis* freezing tolerance and heterosis in freezing tolerance from metabolite composition. Mol Plant 3, 224–235 (2010). 20026477 10.1093/mp/ssp105 PMC2807929

[b22] Gärtner T. *et al.* Improved heterosis prediction by combining information on DNA- and metabolic markers. PLoS ONE 4, e5220 (2009). 19370148 10.1371/journal.pone.0005220 PMC2666157

[b23] Steinfath M. *et al.* Discovering plant metabolic biomarkers for phenotype prediction using an untargeted approach. Plant Biotechnol J 8, 900–911 (2010). 20353402 10.1111/j.1467-7652.2010.00516.x

[b24] Lu B., Cai X. & Jin X. Efficient *indica* and *japonica* rice identification based on the InDel molecular method:its implication in rice breeding and evolutionary research. *Progress in Nature* Science 19, 1241–1252 (2009).

[b25] Sun J. *et al.* The contribution of intersubspecific hybridization to the breeding of super-high-yielding *japonica* rice in northeast China. Theor Appl Genet 125, 1149–1157 (2012). 22660631 10.1007/s00122-012-1901-z

[b26] Hur M. *et al.* A global approach to analysis and interpretation of metabolic data for plant natural product discovery. Nat Prod Rep 30, 565–583 (2013). 23447050 10.1039/c3np20111b PMC3629923

[b27] Eriksson L., Andersson P. L., Johansson E. & Tysklind M. Megavariate analysis of environmental QSAR data. Part I-a basic framework founded on principal component analysis (PCA), partial least squares (PLS), and statistical molecular design (SMD). Mol Divers 10, 169–186 (2006). 16770514 10.1007/s11030-006-9024-6

[b28] Eriksson L., Hermens J. L. M., Johansson E., Verhaar H. J. M. & Wold S. Multivariate analysis of aquatic toxicity data with PLS. Aquat Sci 57, 217–241 (1995).

[b29] Kleessen S. *et al.* Metabolic efficiency underpins performance trade-offs in growth of *Arabidopsis thaliana*. Nat Commun 5, 3537 (2014). 24675291 10.1038/ncomms4537

[b30] Williams W. Heterosis and the genetics of complex characters. Nature 184, 527–530 (1959). 13844942 10.1038/184527a0

[b31] Chen Z. J. Genomic and epigenetic insights into the molecular bases of heterosis. Nat Rev Genet 14, 471–482 (2013). 23752794 10.1038/nrg3503

[b32] Dong X. *et al.* Spatiotemporal distribution of phenolamides and the genetics of natural variation of hydroxycinnamoyl spermidine in rice. Mol Plant 8, 111–121 (2015). 25578276 10.1016/j.molp.2014.11.003

[b33] Edreva A. M., Velikova V. B. & Tsonev T. D. Phenylamides in plants. Russ J Plant Physiol 54, 287–301 (2007).

[b34] Guo D., Sun Y. & Chen Z. Involvement of polyamines in cytoplasmic male sterility of stem mustard(*Brassica juncea* var. *tsatsai*). Plant Growth Regul 41, 33–40 (2003).

[b35] Sulpice R. *et al.* Network analysis of enzyme activities and metabolite levels and their relationship to biomass in a large panel of *Arabidopsis* accessions. Plant Cell 22, 2872–2893 (2010). 20699391 10.1105/tpc.110.076653 PMC2947169

[b36] Riedelsheimer C. *et al.* Genome-wide association mapping of leaf metabolic profiles for dissecting complex traits in maize. Proc Natl Acad Sci USA 109, 8872–8877 (2012). 22615396 10.1073/pnas.1120813109 PMC3384205

[b37] Kusano M., Yang Z., Okazaki Y., Nakabayashi R., Fukushima A. & Saito K. Using metabolomic approaches to explore chemical diversity in rice. Mol Plant 8, 58–67 (2015). 25578272 10.1016/j.molp.2014.11.010

[b38] Kind T. *et al.* FiehnLib: mass spectral and retention index libraries for metabolomics based on quadrupole and time-of-flight gas chromatography/mass spectrometry. Anal Chem 81, 10038–10048 (2009). 19928838 10.1021/ac9019522 PMC2805091

[b39] Qu Z. *et al.* QTL mapping of combining ability and heterosis of agronomic traits in rice backcross recombinant inbred lines and hybrid crosses. PLoS ONE 7, e28463 (2012). 22291881 10.1371/journal.pone.0028463 PMC3266898

