# Supplementary material for: Metabolic prediction of important agronomic traits in hybrid rice (Oryza sativa L.)
Source: Sci Rep. 2016 Feb 24;6:21732. doi: 10.1038/srep21732 (PMC4764848; doi:10.1038/srep21732)
Supplement: Supplementary Dataset 1 [file srep21732-s1.doc]

# Metabolic prediction of important agronomic traits in hybrid rice (*Oryza sativa* L.)

Zhiwu Dan^1,2^, Jun Hu^1,2^, Wei Zhou^1,2^, Guoxin Yao^1,2^, Renshan Zhu^1,2^, Yingguo Zhu^1,2,*^, Wenchao Huang^1,2,*^.

| Table S1. Information of the eighteen rice inbred lines. | | | |
| --- | --- | --- | --- |
| Cultivar Name | F_i_ | Origin | Groups |
| R465 | 1.00 | India | *Indica* |
| YB | 0.95 | Hubei, China |
| Mianhui725 | 0.92 | Mianyang, Sichuan, China |
| 610234 | 0.88 | Wuhan, Hubei, China |
| 9311K | 0.82 | Chengdu, Sichuan, China |
| Qianlijing | 0.70 | Ya'an, Sichuan, China |
| W1394 | 0.55 | Nanjing, Jiangsu, China |
| R4115 | 0.42 | Hunan, China |
| C418 | 0.39 | Liaoning, China |
| W1384 | 0.33 | Nanjing, Jiangsu, China | *Japonica* |
| W1383 | 0.30 | Nanjing, Jiangsu, China |
| W1392 | 0.20 | Nanjing, Jiangsu, China |
| W1390 | 0.18 | Nanjing, Jiangsu, China |
| Liaoxing1 | 0.12 | Shenyang, Liaoning, China |
| JR2 | 0.11 | Yunnan, China |
| 110080 | 0.09 | Jiangsu, China |
| Wuyunjing8 | 0.02 | Wujin, Jiangsu, China |
| Balilla | 0.00 | Italy |
|  |  |  |  |
| Cultivar Name | YPP(g) | MSPH(cm) | HD(d) |
| R465 | 49.518756195767 | 133.204656084656 | 86.333333333333 |
| YB | 41.374628343615 | 119.162824675325 | 81.000000000000 |
| Mianhui725 | 40.719540632937 | 133.898947811448 | 90.000000000000 |
| 610234 | 34.130734966667 | 121.214444444444 | 91.666666666667 |
| 9311K | 41.707307884259 | 132.957407407407 | 102.333333333333 |
| Qianlijing | 27.948534619048 | 118.719523809524 | 77.666666666667 |
| W1394 | 26.616562244444 | 125.123809523810 | 106.333333333333 |
| R4115 | 30.642791133333 | 128.519047619048 | 79.000000000000 |
| C418 | 16.283967016667 | 127.711666666667 | 84.333333333333 |
| W1384 | 37.795407922222 | 137.918333333333 | 98.666666666667 |
| W1383 | 13.881220527778 | 128.194047619048 | 88.333333333333 |
| W1392 | 27.853152611111 | 121.463888888889 | 101.666666666667 |
| W1390 | 29.600970237013 | 127.885151515152 | 103.000000000000 |
| Liaoxing1 | 38.552647583333 | 144.790000000000 | 72.000000000000 |
| JR2 | 32.531728285185 | 147.591481481482 | 93.333333333333 |
| 110080 | 12.560646950000 | 94.776190476191 | 92.666666666667 |
| Wuyunjing8 | 15.913897027778 | 89.205555555556 | 94.000000000000 |
| Balilla | 20.079962910053 | 93.266296296296 | 72.333333333333 |
|  |  |  |  |
| Cultivar Name | GCA-YPP | GCA-MSPH | GCA-HD |
| R465 | 1.740780319069 | 10.241115371237 | -0.165023112481 |
| YB | 13.183430850699 | 9.698283009336 | -0.873579262213 |
| Mianhui725 | -0.518384073929 | 6.171444783687 | 0.966290018832 |
| 610234 | 4.050883700466 | 5.215925193850 | 3.198613251156 |
| 9311K | 6.536479832049 | 7.550729859871 | 7.538185443669 |
| Qianlijing | 11.623179632265 | 0.607164190280 | -8.401765536723 |
| W1394 | -6.170925187822 | 3.017951189739 | 9.824875877418 |
| R4115 | 4.254711375803 | -1.799943725803 | -9.452010634762 |
| C418 | 0.930751586779 | -4.314471072095 | -7.059853772017 |
| W1384 | -4.681375543582 | 3.967492541634 | 8.273479561316 |
| W1383 | -2.068451011410 | 2.189291549115 | 5.375020177563 |
| W1392 | -4.873485194875 | 1.832784680901 | 8.087817796610 |
| W1390 | -5.398758921756 | -0.273669143053 | 7.785734463277 |
| Liaoxing1 | -4.896040211182 | -13.704667820302 | -12.412182203390 |
| JR2 | 11.952713261072 | 12.557550939036 | -4.523608971067 |
| 110080 | -5.049247416453 | -9.966211031317 | 2.703663756206 |
| Wuyunjing8 | -11.389003340744 | -14.937637489259 | 6.016795069337 |
| Balilla | -9.670568853511 | -18.528709419573 | -16.170073617531 |
| F_i_ is the proportion of *indica*-*japonica* content which is estimated with the InDel marker method. YPP=Yield per plant; MSPH=Maturation stage plant height; HD=Heading date; GCA=General combining ability. | | | |

Table S2. Correlation between hybrid phenotypes and relative heterosis, specific combining ability.

| YPP(g) | LPH-YPP | MPH-YPP | BPH-YPP | YPP-SCA |
| --- | --- | --- | --- | --- |
| Pearson Correlation | 0.530^†^ | 0.717^†^ | 0.732^†^ | 0.774^†^ |
| Sig. (2-tailed) | 0.000 | 0.000 | 0.000 | 0.000 |
| N | 291 | 291 | 291 | 291 |
| MSPH(cm) | LPH-MSPH | MPH-MSPH | BPH-MSPH | MSPH-SCA |
| Pearson Correlation | 0.374^†^ | 0.717^†^ | 0.824^†^ | 0.720^†^ |
| Sig. (2-tailed) | 0.000 | 0.000 | 0.000 | 0.000 |
| N | 295 | 295 | 295 | 295 |
| HD(d) | LPH-HD | MPH-HD | BPH-HD | HD-SCA |
| Pearson Correlation | -0.853^†^ | -0.879^†^ | -0.753^†^ | -0.677^†^ |
| Sig. (2-tailed) | 0.000 | 0.000 | 0.000 | 0.000 |
| N | 295 | 295 | 295 | 295 |

YPP=Yield per plant, MSPH=Maturation stage plant height, HD=Heading date, LPH=Low-parent heterosis, Mid-parent heterosis, BPH=better-parent heterosis, SCA=Specific combining ability. ^†^. Correlation is significant at the 0.01 level (2-tailed).

Table S3. Correlation between parental phenotypes and hybrid phenotypes.

| Traits |  | Mean-YPP(g)^*^ | Minus-YPP(g) | Divide-YPP(g) |
| --- | --- | --- | --- | --- |
| YPP(g) | Pearson Correlation | 0.297^†^ | -0.03 | -0.05 |
|  | Sig. (2-tailed) | 0.00 | 0.59 | 0.36 |
|  | N | 291 | 291 | 291 |
|  | | Mean-MSPH  (cm) | Minus-MSPH  (cm) | Divide-MSPH  (cm) |
| MSPH(cm) | Pearson Correlation | 0.470^†^ | 0.04 | -0.02 |
|  | Sig. (2-tailed) | 0.00 | 0.46 | 0.77 |
|  | N | 295 | 295 | 295 |
|  | | Mean-HD(d) | Minus-HD(d) | Divide-HD(d) |
| HD(d) | Pearson Correlation | 0.714^†^ | 0.02 | 0.01 |
|  | Sig. (2-tailed) | 0.00 | 0.69 | 0.90 |
|  | N | 295 | 295 | 295 |

*. Means of parental yield per plant. †. Correlation is significant at the 0.01 level (2-tailed). YPP=Yield per plant, MSPH=Maturation stage plant height, HD=Heading date, Mean=Mean values of corresponding parents, Minus=Differences of values between corresponding parents. Divide=Ratios of corresponding parents’ values.

| Table S4 Squared Euclidean Distance basing on parental lines metabolite profiling | | | | | | |
| --- | --- | --- | --- | --- | --- | --- |
| Case | 1:YB | 2:R4115 | 3:Qianlijing | 4:W1383 | 5:W1394 | 6:610234 |
| 1:YB | 0.000 | 374.845 | 369.624 | 408.281 | 654.717 | 336.159 |
| 2:R4115 | 374.845 | 0.000 | 338.557 | 332.718 | 588.096 | 468.068 |
| 3:Qianlijing | 369.624 | 338.557 | 0.000 | 281.626 | 486.486 | 299.625 |
| 4:W1383 | 408.281 | 332.718 | 281.626 | 0.000 | 200.102 | 248.409 |
| 5:W1394 | 654.717 | 588.096 | 486.486 | 200.102 | 0.000 | 400.503 |
| 6:610234 | 336.159 | 468.068 | 299.625 | 248.409 | 400.503 | 0.000 |
| 7:Mianhui725 | 277.985 | 404.713 | 362.461 | 344.283 | 599.348 | 317.407 |
| 8:Liaoxing1 | 545.867 | 429.873 | 353.061 | 325.777 | 449.938 | 449.431 |
| 9:Balilla | 661.183 | 610.102 | 461.063 | 382.699 | 482.874 | 528.177 |
| 10:9311K | 386.989 | 487.778 | 458.249 | 314.956 | 453.620 | 251.927 |
| 11:W1390 | 525.349 | 380.185 | 367.550 | 187.960 | 257.954 | 389.922 |
| 12:R465 | 491.502 | 643.661 | 353.976 | 268.269 | 309.472 | 246.725 |
| 13:W1392 | 714.027 | 645.165 | 625.321 | 305.988 | 366.738 | 490.156 |
| 14:110080 | 591.107 | 428.591 | 448.462 | 192.350 | 272.640 | 407.780 |
| 15:W1384 | 361.921 | 295.774 | 352.932 | 180.016 | 352.285 | 365.181 |
| 16:JR2 | 417.553 | 263.634 | 297.017 | 233.862 | 413.109 | 401.481 |
| 17:C418 | 454.902 | 308.767 | 385.114 | 273.450 | 468.723 | 440.454 |
| 18:Wuyunjing8 | 597.082 | 494.000 | 472.987 | 262.867 | 318.770 | 476.802 |
|  |  |  |  |  |  |  |
| Case | 7:Mianhui725 | 8:Liaoxing1 | 9:Balilla | 10:9311K | 11:W1390 | 12:R465 |
| 1:YB | 277.985 | 545.867 | 661.183 | 386.989 | 525.349 | 491.502 |
| 2:R4115 | 404.713 | 429.873 | 610.102 | 487.778 | 380.185 | 643.661 |
| 3:Qianlijing | 362.461 | 353.061 | 461.063 | 458.249 | 367.550 | 353.976 |
| 4:W1383 | 344.283 | 325.777 | 382.699 | 314.956 | 187.960 | 268.269 |
| 5:W1394 | 599.348 | 449.938 | 482.874 | 453.620 | 257.954 | 309.472 |
| 6:610234 | 317.407 | 449.431 | 528.177 | 251.927 | 389.922 | 246.725 |
| 7:Mianhui725 | 0.000 | 417.728 | 450.575 | 370.805 | 327.575 | 395.394 |
| 8:Liaoxing1 | 417.728 | 0.000 | 253.979 | 610.180 | 308.272 | 420.776 |
| 9:Balilla | 450.575 | 253.979 | 0.000 | 647.351 | 298.739 | 394.793 |
| 10:9311K | 370.805 | 610.180 | 647.351 | 0.000 | 454.152 | 340.433 |
| 11:W1390 | 327.575 | 308.272 | 298.739 | 454.152 | 0.000 | 295.546 |
| 12:R465 | 395.394 | 420.776 | 394.793 | 340.433 | 295.546 | 0.000 |
| 13:W1392 | 506.708 | 458.740 | 333.580 | 507.609 | 248.433 | 401.575 |
| 14:110080 | 443.453 | 274.494 | 251.566 | 397.439 | 212.039 | 332.619 |
| 15:W1384 | 344.251 | 343.461 | 417.722 | 387.305 | 262.282 | 426.587 |
| 16:JR2 | 330.646 | 260.881 | 287.629 | 403.912 | 233.401 | 396.270 |
| 17:C418 | 346.963 | 206.562 | 350.116 | 542.925 | 268.145 | 441.906 |
| 18:Wuyunjing8 | 447.819 | 336.348 | 300.587 | 451.890 | 242.770 | 339.742 |
|  |  |  |  |  |  |  |
| Case | 13:W1392 | 14:110080 | 15:W1384 | 16:JR2 | 17:C418 | 18:Wuyunjing8 |
| 1:YB | 714.027 | 591.107 | 361.921 | 417.553 | 454.902 | 597.082 |
| 2:R4115 | 645.165 | 428.591 | 295.774 | 263.634 | 308.767 | 494.000 |
| 3:Qianlijing | 625.321 | 448.462 | 352.932 | 297.017 | 385.114 | 472.987 |
| 4:W1383 | 305.988 | 192.350 | 180.016 | 233.862 | 273.450 | 262.867 |
| 5:W1394 | 366.738 | 272.640 | 352.285 | 413.109 | 468.723 | 318.770 |
| 6:610234 | 490.156 | 407.780 | 365.181 | 401.481 | 440.454 | 476.802 |
| 7:Mianhui725 | 506.708 | 443.453 | 344.251 | 330.646 | 346.963 | 447.819 |
| 8:Liaoxing1 | 458.740 | 274.494 | 343.461 | 260.881 | 206.562 | 336.348 |
| 9:Balilla | 333.580 | 251.566 | 417.722 | 287.629 | 350.116 | 300.587 |
| 10:9311K | 507.609 | 397.439 | 387.305 | 403.912 | 542.925 | 451.890 |
| 11:W1390 | 248.433 | 212.039 | 262.282 | 233.401 | 268.145 | 242.770 |
| 12:R465 | 401.575 | 332.619 | 426.587 | 396.270 | 441.906 | 339.742 |
| 13:W1392 | 0.000 | 167.861 | 397.448 | 417.768 | 424.417 | 226.022 |
| 14:110080 | 167.861 | 0.000 | 246.413 | 257.614 | 261.759 | 128.142 |
| 15:W1384 | 397.448 | 246.413 | 0.000 | 247.810 | 267.130 | 312.738 |
| 16:JR2 | 417.768 | 257.614 | 247.810 | 0.000 | 282.628 | 273.392 |
| 17:C418 | 424.417 | 261.759 | 267.130 | 282.628 | 0.000 | 366.579 |
| 18:Wuyunjing8 | 226.022 | 128.142 | 312.738 | 273.392 | 366.579 | 0.000 |

Table S5. Correlation between parental genetic distance and hybrid performance.

| Traits | N | Pearson Correlation | Sig. (2-tailed) |
| --- | --- | --- | --- |
| YPP(g) | 291 | 0.213^†^ | 0.000 |
| MSPH(cm) | 295 | 0.271^†^ | 0.000 |
| HD(d) | 295 | 0.013 | 0.816 |
| LPH-YPP | 291 | -0.036 | 0.538 |
| LPH-MSPH | 295 | 0.260^†^ | 0.000 |
| LPH-HD | 295 | -0.006 | 0.914 |
| MPH-YPP | 291 | -0.032 | 0.590 |
| MPH-MSPH | 295 | 0.342^†^ | 0.000 |
| MPH-HD | 295 | -0.109 | 0.061 |
| BPH-YPP | 291 | -0.026 | 0.664 |
| BPH-MSPH | 295 | 0.323^†^ | 0.000 |
| BPH-HD | 295 | -0.221^†^ | 0.000 |
| YPP-SCA | 291 | 0.114 | 0.053 |
| MSPH-SCA | 295 | 0.332^†^ | 0.000 |
| HD-SCA | 295 | -0.191^†^ | 0.001 |

†. Correlation is significant at the 0.01 level (2-tailed). YPP=Yield per plant, MSPH=Maturation stage plant height, HD=Heading date, LPH=Relative low-parent heterosis, MPH=Relative mid-parent heterosis, BPH=Relative better-parent heterosis, SCA=Specific combining ability.

Table S6. Stepwise linear regression with parental relative metabolite levels and hybrid performance.

|  | Dependent Variable | Number of predictors | Adjusted R^2^ |
| --- | --- | --- | --- |
|  | YPP(g) | 6 | 0.417 |
|  | MSPH(cm) | 6 | 0.532 |
|  | HD(d) | 6 | 0.588 |
|  | LPH-YPP | 7 | 0.513 |
|  | LPH-MSPH | 2 | 0.375 |
|  | LPH-HD | 4 | 0.331 |
|  | MPH-YPP | 6 | 0.434 |
| Mean | MPH-MSPH | 5 | 0.419 |
|  | MPH-HD | 4 | 0.250 |
|  | BPH-YPP | 5 | 0.348 |
|  | BPH-MSPH | 5 | 0.463 |
|  | BPH-HD | 5 | 0.177 |
|  | YPP-SCA | - | - |
|  | MSPH-SCA | - | - |
|  | HD-SCA | - | - |
|  | Dependent Variable | Number of predictors | Adjusted R^2^ |
| Minus | YPP(g) | 3 | 0.074 |
|  | MSPH(cm) | 3 | 0.067 |
|  | HD(d) | 4 | 0.152 |
|  | LPH-YPP | 3 | 0.092 |
|  | LPH-MSPH | 3 | 0.136 |
|  | LPH-HD | - | - |
|  | MPH-YPP | 4 | 0.077 |
|  | MPH-MSPH | 3 | 0.084 |
|  | MPH-HD | 3 | 0.123 |
|  | BPH-YPP | 2 | 0.042 |
|  | BPH-MSPH | 3 | 0.062 |
|  | BPH-HD | 4 | 0.144 |
|  | YPP-SCA | - | - |
|  | MSPH-SCA | - | - |
|  | HD-SCA | - | - |
|  | Dependent Variable | Number of predictors | Adjusted R^2^ |
| Divide | YPP(g) | 7 | 0.204 |
|  | MSPH(cm) | 22 | 0.675 |
|  | HD(d) | 21 | 0.677 |
|  | LPH-YPP | 3 | 0.145 |
|  | LPH-MSPH | 12 | 0.543 |
|  | LPH-HD | 7 | 0.389 |
|  | MPH-YPP | 2 | 0.114 |
|  | MPH-MSPH | 10 | 0.467 |
|  | MPH-HD | 11 | 0.409 |
|  | BPH-YPP | 6 | 0.179 |
|  | BPH-MSPH | 14 | 0.540 |
|  | BPH-HD | 22 | 0.571 |
|  | YPP-SCA | 1 | 0.033 |
|  | MSPH-SCA | 13 | 0.399 |
|  | HD-SCA | 5 | 0.296 |

YPP=Yield per plant, MSPH=Maturation stage plant height, HD=Heading date, LPH=Relative low-parent heterosis, MPH=Relative mid-parent heterosis, BPH=Relative better-parent heterosis, SCA=Specific combining ability. Mean=Mean values of corresponding parents, Minus=Differences of values between corresponding parents. Divide=Ratios of corresponding parents’ values.

Table S8. Predictive abilities of reciprocal hybrids.

| Traits | Predictive ability A | Predictive ability A'^*^ |
| --- | --- | --- |
| YPP | 0.930 | 0.919 |
| MSPH | 0.953 | 0.949 |
| HD | 0.978 | 0.975 |
| LPH-YPP | 0.929 | 0.935 |
| LPH-MSPH | 0.952 | 0.956 |
| LPH-HD | 0.968 | 0.959 |
| MPH-YPP | 0.924 | 0.921 |
| MPH-MSPH | 0.939 | 0.938 |
| MPH-HD | 0.960 | 0.948 |
| BPH-YPP | 0.925 | 0.911 |
| BPH-MSPH | 0.948 | 0.949 |
| BPH-HD | 0.936 | 0.928 |
| SCA-YPP | 0.853 | 0.866 |
| SCA-MSPH | 0.878 | 0.893 |
| SCA-HD | 0.947 | 0.933 |

^*^, Predictive ability of corresponding reciprocal hybrid group. YPP=Yield per plant, MSPH=Maturation stage plant height, HD=Heading date, LPH=Relative low-parent heterosis, MPH=Relative mid-parent heterosis, BPH=Relative better-parent heterosis, SCA=Specific combining ability.

| Table S9 Overlapped predictive variables for different traits. | | |
| --- | --- | --- |
| Divide-Metabolite-YPP | Divide-Metabolite-MSPH | Divide-Metabolite-HD |
| - | - | Analyte1 |
| Analyte3 | Analyte3 | - |
| Analyte4 | Analyte8 | - |
| Analyte5 | - | Analyte5 |
| - | Analyte11 | - |
| Analyte7 | - | Analyte7 |
| Analyte12 | - | Analyte12 |
| - | Analyte13 | - |
| - | Analyte14 | Analyte14 |
| Analyte15 | - | Analyte15 |
| Analyte16 | - | Analyte16 |
| Analyte19 | - | Analyte19 |
| Analyte21 | - | - |
| Analyte23 | - | - |
| Analyte24 | - | - |
| Analyte25 | - | Analyte25 |
| Analyte27 | - | - |
| 2hydroxypyridine1 | - | - |
| Analyte28 | - | - |
| Analyte30 | - | - |
| Analyte31 | - | - |
| Analyte32 | Analyte32 | - |
| Analyte34 | - | Analyte34 |
| Analyte36 | - | - |
| - | Analyte33 | - |
| - | - | lacticacid1 |
| - | lacticacid2 | - |
| - | Analyte39 | - |
| - | Analyte41 | Analyte41 |
| - | - | Analyte44 |
| Analyte 38 | - | - |
| glycolicacid1 | - | - |
| glycolicacid2 | - | - |
| Analyte45 | - | - |
| - | Analyte42 | - |
| alanine1 | alanine1 | alanine1 |
| Analyte49 | - | Analyte49 |
| Analyte51 | - | Analyte51 |
| hydroxylamine | - | - |
| Analyte48 | - | - |
| Analyte50 | Analyte50 | - |
| - | - | 2FuroicAcid |
| Lactamide2 | - | Lactamide2 |
| - | oxalicacid | - |
| 3Hydroxypropionicacid1 | - | 3Hydroxypropionicacid1 |
| pcresol | pcresol | pcresol |
| Analyte54 | - | - |
| 3hydroxybutyricacid | - | - |
| Analyte56 | - | Analyte56 |
| - | Analyte59 | Analyte59 |
| NMethylDLalanine | - | NMethylDLalanine |
| sulfuricacid | - | - |
| Analyte58 | - | - |
| 2Ketovalericacid1 | - | - |
| MethylPhosphate | MethylPhosphate | - |
| - | Isoleucine1 | - |
| Analyte61 | - | - |
| Analyte62 | - | Analyte62 |
| Analyte63 | - | Analyte63 |
| Analyte64 | Analyte64 | Analyte64 |
| malonicacid1 | - | malonicacid1 |
| - | Analyte67 | - |
| - | - | Analyte70 |
| Analyte72 | - | Analyte72 |
| Analyte68 | - | - |
| Analyte69 | - | - |
| 1Aminocyclopropanecarboxylicacid | - | 1Aminocyclopropanecarboxylicacid |
| Methylmalonicacid1 | - | - |
| - | Analyte74 | Analyte74 |
| valine2 | - | valine2 |
| 10Hydroxydecanoicacid | - | - |
| - | 15Anhydroglucitol | - |
| - | 2425dihydrolanosterol2 | - |
| 1Indanol | 1Indanol | 1Indanol |
| 2425dihydrolanosterol1 | - | 2425dihydrolanosterol1 |
| 24diaminobutyricacid4 | 24diaminobutyricacid4 | 24diaminobutyricacid1 |
| 2hydroxy3isopropylbutanedioicacid | 2hydroxy3isopropylbutanedioicacid | - |
| - | 2Deoxyerythritol | - |
| - | - | 2aminophenol2 |
| 2ketoadipate3 | 2ketoadipate3 | 2ketoadipate3 |
| - | - | 2R3S2hydroxy3isopropylbutanedioicacid |
| - | 35Dihydroxyphenylglycine2 | 35Dihydroxyphenylglycine2 |
| - | 36AnhydroDgalactose2 | 36AnhydroDgalactose2 |
| - | 2methylfumarate2 | - |
| 3712Trihydroxycoprostane1 | - | 3712Trihydroxycoprostane1 |
| 3Aminoisobutyricacid1 | - | 3Aminoisobutyricacid1 |
| - | 3Cyanoalanine | 3Cyanoalanine |
| - | - | 3hydroxybenzylalcohol |
| 3hydroxyLproline2 | - | - |
| - | 3Indolepyruvicacid | 3Indolepyruvicacid |
| - | - | 3MethylglutaricAcid |
| - | - | 4Acetylbutyricacid1 |
| - | 4hydroxy3methoxybenzoicaci | - |
| - | - | 4aminobutyricacid1 |
| 4aminobutyricacid2 | - | - |
| 4Cholesten3one3 | - | 4Cholesten3one3 |
| - | - | 4Androstene317dione2 |
| - | - | 4Hydroxyquinazoline |
| - | 5Dihydrocortisone1 | 5Dihydrocortisone1 |
| 5Hydroxyindole2carboxylicacid2 | - | 5Hydroxyindole2carboxylicacid2 |
| 5Methoxytryptamine1 | - | - |
| - | 6deoxyDglucose1 | 6deoxyDglucose1 |
| AconiticAcid | AconiticAcid | AconiticAcid |
| 6phosphogluconicacid | - | - |
| - | Adenosine5monophosphate | Adenosine5monophosphate |
| Adipamide3 | Adipamide3 | - |
| - | - | Adipamide2 |
| Aldosterone2 | - | Aldosterone2 |
| - | alphaSantonin11 | alphaSantonin11 |
| - | alphaSantonin12 | alphaSantonin12 |
| Aminomalonicacid | - | Aminomalonicacid |
| allose1 | allose1 | - |
| - | alphaDglucosamine1phosphate1 | - |
| Allantoicacid1 | - | - |
| Allantoicacid22 | - | - |
| alphaAminoadipicacid | - | - |
| alphaDglucosamine1phosphate2 | - | - |
| alphaketoglutaricacid | - | - |
| - | Analyte164 | Analyte164 |
| Analyte169 | Analyte169 | Analyte169 |
| Analyte165 | - | - |
| - | Analyte170 | Analyte170 |
| - | - | Analyte172 |
| Analyte171 | - | - |
| Analyte1091 | - | - |
| Analyte176 | - | Analyte176 |
| - | Analyte178 | Analyte178 |
| - | Analyte179 | Analyte179 |
| Analyte180 | - | - |
| Analyte181 | Analyte181 | - |
| - | Analyte185 | Analyte185 |
| Analyte182 | - | - |
| Analyte188 | - | Analyte188 |
| Analyte189 | - | Analyte189 |
| Analyte191 | - | - |
| Analyte192 | Analyte192 | Analyte192 |
| Analyte194 | - | Analyte194 |
| - | - | Analyte196 |
| Analyte197 | - | - |
| Analyte75 | - | Analyte75 |
| Analyte76 | - | Analyte76 |
| Analyte78 | - | Analyte78 |
| - | Analyte82 | - |
| - | Analyte84 | - |
| - | Analyte85 | Analyte85 |
| Analyte89 | - | - |
| - | - | Analyte357 |
| - | - | Analyte88 |
| Analyte92 | Analyte92 | - |
| - | Analyte97 | - |
| - | Analyte102 | Analyte102 |
| - | Analyte104 | Analyte104 |
| - | Analyte105 | Analyte105 |
| Analyte107 | - | Analyte107 |
| Analyte688 | Analyte688 | - |
| - | Analyte112 | - |
| - | - | Analyte111 |
| - | Analyte116 | Analyte116 |
| - | Analyte113 | Analyte113 |
| Analyte118 | - | Analyte118 |
| - | Analyte115 | - |
| - | - | Analyte120 |
| Analyte121 | - | Analyte121 |
| - | Analyte128 | - |
| - | Analyte131 | Analyte131 |
| - | Analyte133 | Analyte133 |
| Analyte123 | - | - |
| Analyte127 | - | - |
| - | Analyte134 | - |
| - | Analyte135 | - |
| Analyte136 | - | - |
| Analyte138 | - | Analyte138 |
| Analyte139 | Analyte139 | Analyte139 |
| Analyte141 | Analyte141 | - |
| Analyte142 | - | - |
| Analyte143 | Analyte143 | - |
| - | Analyte144 | Analyte144 |
| - | - | Analyte146 |
| Analyte147 | Analyte147 | - |
| - | - | Analyte148 |
| - | - | Analyte150 |
| - | Analyte151 | - |
| - | Analyte153 | - |
| Analyte154 | - | Analyte154 |
| - | Analyte157 | Analyte157 |
| - | asparagine4 | - |
| Analyte159 | - | - |
| Analyte160 | - | Analyte160 |
| Analyte162 | - | Analyte162 |
| asparagine1 | - | asparagine1 |
| - | - | asparagine3 |
| - | asparagine5 | - |
| - | asparticacid1 | - |
| - | betaAlanine22 | - |
| - | betaGlutamicacid1 | - |
| Arachidicacid | - | - |
| Atropine1 | - | Atropine1 |
| ascorbate1 | - | - |
| - | Biuret1 | - |
| - | Biuret2 | - |
| benzoicacid | - | benzoicacid |
| Atropine2 | - | - |
| betahydroxypyruvate | - | betahydroxypyruvate |
| betaMannosylglycerate2 | - | betaMannosylglycerate2 |
| butyraldehyde1 | - | butyraldehyde1 |
| - | Citraconicacid4 | Citraconicacid4 |
| - | citricacid | citricacid |
| betaAlanine21 | - | - |
| - | - | cellobiose1 |
| - | - | cellobiose2 |
| - | Dgalacturonicacid21 | - |
| - | cytidinemonophosphatedegrprod | cytidinemonophosphatedegrprod |
| Cerotinicacid | - | Cerotinicacid |
| cisgondoicacid | - | cisgondoicacid |
| carbamoylasparticacid21 | carbamoylasparticacid21 | - |
| carbamoylasparticacid22 | carbamoylasparticacid22 | - |
| - | - | citrulline11 |
| - | - | citrulline2 |
| CarbobenzyloxyLleucinedegr2 | - | - |
| Cortexolone2 | - | Cortexolone2 |
| cytidine5monophosphate | - | - |
| - | dGlucoheptose11 | - |
| - | dGlucoheptose12 | - |
| - | Digitoxose1 | - |
| - | Diglycerol12 | Diglycerol12 |
| Erythrose2 | Erythrose2 | - |
| - | ferulicacid | - |
| - | - | dGlucoheptose2 |
| DehydroascorbicAcid11 | - | - |
| Dgalacturonicacid22 | Dgalacturonicacid22 | - |
| - | - | DGlycericacid |
| Diglycerol11 | - | - |
| - | - | Digitoxose2 |
| Dihydroxyacetone1 | - | - |
| Dihydroxyacetone2 | - | - |
| - | Fructose26biphosphatedegrprod2 | - |
| - | fructose6phosphate | - |
| - | - | Dihydrocarveol |
| Dioctylphthalate | Dioctylphthalate | Dioctylphthalate |
| - | - | DLAnabasine1 |
| Ergosterol | - | Ergosterol |
| - | fucose1 | - |
| - | Galactinol11 | Galactinol11 |
| - | Gentiobiose22 | Gentiobiose22 |
| - | Glucoheptonicacid3 | - |
| - | gluconicacid2 | - |
| - | - | Galactinol12 |
| fructose2 | fructose2 | - |
| Glucosaminicacid2 | Glucosaminicacid2 | Glucosaminicacid2 |
| fumaricacid | - | - |
| - | - | Glucoheptonicacid11 |
| - | glucose6phosphate11 | - |
| - | glucose6phosphate12 | - |
| gluconicacid1 | - | - |
| - | glucose6phosphate2 | - |
| glutamine1 | glutamine1 | glutamine1 |
| - | glutamine2 | - |
| Glucosaminicacid1 | - | - |
| - | - | GlutaricAcid2 |
| - | - | guanosine |
| glycocyamine1 | - | - |
| glycocyamine3 | glycocyamine3 | - |
| glypro | glypro | - |
| histidine2 | - | histidine2 |
| hydroxyurea | - | - |
| - | lactitol | lactitol |
| - | lauricacid | - |
| - | - | Isoleucine2 |
| lactose2 | - | - |
| - | - | Lcysteine |
| - | Lhomoserine1 | Lhomoserine1 |
| - | - | Lignocericacid |
| linoleicacid | linoleicacid | linoleicacid |
| - | linolenicacid | - |
| leucine | - | - |
| - | lysine | - |
| LMalicacid | - | LMalicacid |
| LThreose2 | - | LThreose2 |
| - | Lyxonicacid14lactone | - |
| Linoleicacidmethylester1 | - | - |
| Linoleicacidmethylester2 | - | - |
| - | maleicacid | maleicacid |
| - | malonicacid21 | - |
| - | - | MALONAMIDE2 |
| - | - | MALONAMIDE4 |
| - | MethylPalmitoleate | - |
| - | Monostearin | - |
| NepsilonTrimethyllysine | NepsilonTrimethyllysine | - |
| - | mucicacid | - |
| - | - | melibiose11 |
| - | - | methionine1 |
| - | NAcetylbetaalanine2 | NAcetylbetaalanine2 |
| - | NacetylLasparticacid1 | NacetylLasparticacid1 |
| - | Nethylmaleamicacid31 | Nethylmaleamicacid31 |
| - | - | Nethylmaleamicacid32 |
| - | oleicacid | oleicacid |
| OPhosphorylethanolamine | OPhosphorylethanolamine | OPhosphorylethanolamine |
| ornithine2 | - | - |
| Oroticacid | Oroticacid | - |
| oxamicacid | - | oxamicacid |
| - | oxamide | oxamide |
| - | oxoproline | oxoproline |
| phenylalanine1 | - | phenylalanine1 |
| proline | - | - |
| ProstaglandinE22 | ProstaglandinE22 | - |
| Pyrrole2CarboxylicAcid | - | - |
| - | palmiticacid | - |
| - | pantothenicacid | pantothenicacid |
| - | Phenylaceticacid | Phenylaceticacid |
| - | Phytanicacid | Phytanicacid |
| - | Pipecolinicacid | - |
| - | Purineriboside1 | Purineriboside1 |
| - | - | phenylalanine2 |
| - | - | PhenylbetaDglucopyranoside |
| - | - | phosphomycin |
| - | - | Phytol |
| - | - | progesterone |
| - | - | Purineriboside2 |
| ribulose5phosphate1 | - | ribulose5phosphate1 |
| - | raffinose | raffinose |
| Saccharicacid1 | Saccharicacid1 | Saccharicacid1 |
| - | Saccharicacid2 | Saccharicacid2 |
| Sitosterol | - | Sitosterol |
| spermidine11 | - | spermidine11 |
| Stigmasterol2 | - | Stigmasterol2 |
| - | serine11 | - |
| - | sorbitol | sorbitol |
| - | stearicacid | - |
| - | - | Stigmasterol1 |
| - | - | succinicacid |
| - | - | Tartronicacid |
| Taxifolin | - | Taxifolin |
| Threonicacid2 | - | - |
| Triacontanoicacidmethylester1 | Triacontanoicacidmethylester1 | Triacontanoicacidmethylester1 |
| Triacontanoicacidmethylester2 | Triacontanoicacidmethylester2 | - |
| tryptophan1 | tryptophan1 | tryptophan1 |
| tyrosine1 | tyrosine1 | - |
| - | Threonicacid3 | Threonicacid3 |
| - | thymine | - |
| - | toluenesulfonicacid | toluenesulfonicacid |
| - | trehalose | - |
| - | - | trans35Dimethoxy4hydroxycinnamaldehyde2 |
| Analyte 101 | - | - |
| Analyte 103 | - | - |
| - | Analyte 174 | Analyte 174 |
| - | Analyte 117 | - |
| Analyte 129 | - | - |
| - | Analyte 130 | Analyte 130 |
| Analyte 156 | - | Analyte 156 |
| - | Analyte 175 | Analyte 175 |
| Analyte 73 | - | Analyte 73 |
| Analyte 79 | - | - |
| - | Analyte 83 | - |
| Analyte 87 | - | Analyte 87 |
| uracil5carboxylicacid | - | - |
| Zymosterol2 | - | Zymosterol2 |
| Metabolites in red color indicate overlapped predictive variables for yield per plant and heading date. Metabolites in purple indicate overlapped predictive variables for yield per plant and maturation stage plant height. Metabolites in blue color indicate overlapped predictive variables for maturation stage plant height and heading date. Metabolites in green color indicate overlapped predictive variables for yield per plant, maturation stage plant height and heading date. The number of overlapped metabolite are: 18(YPP,HD&MSPH); 83(YPP&HD);44(YPP&MSPH);73(HD&MSPH).YPP=Yield per plant, MSPH=Maturation stage plant height, HD=Heading date. | | |
|
|
|
|
|
|
|
|

Table S10. Correlation between parental relative metabolite levels and hybrid phenotypes.

| Metabolites^‡^ |  | YPP(g) | MSPH(cm) | HD(d) |
| --- | --- | --- | --- | --- |
| alanine 1 | Pearson Correlation | 0.149^*^ | 0.323^†^ | 0.197^†^ |
|  | Sig. (2-tailed) | 0.011 | 0.000 | 0.001 |
|  | N | 291 | 295 | 295 |
| Analyte 64 | Pearson Correlation | -0.098 | 0.080 | 0.125^*^ |
|  | Sig. (2-tailed) | 0.095 | 0.171 | 0.032 |
|  | N | 291 | 295 | 295 |
| 2-ketoadipate 3 | Pearson Correlation | -0.094 | 0.092 | 0.135^*^ |
|  | Sig. (2-tailed) | 0.111 | 0.117 | 0.020 |
|  | N | 291 | 295 | 295 |
| 1-Indanol | Pearson Correlation | -0.097 | -0.145^*^ | -0.161^†^ |
|  | Sig. (2-tailed) | 0.100 | 0.013 | 0.006 |
|  | N | 291 | 295 | 295 |
| 2,4-diaminobutyric acid 1 | Pearson Correlation | -0.067 | 0.006 | 0.123^*^ |
|  | Sig. (2-tailed) | 0.256 | 0.924 | 0.035 |
|  | N | 291 | 295 | 295 |
| Aconitic Acid | Pearson Correlation | -0.095 | 0.096 | 0.138^*^ |
|  | Sig. (2-tailed) | 0.105 | 0.101 | 0.018 |
|  | N | 291 | 295 | 295 |
| glutamine 1 | Pearson Correlation | 0.236^†^ | 0.092 | -0.126^*^ |
|  | Sig. (2-tailed) | 0.000 | 0.113 | 0.031 |
|  | N | 291 | 295 | 295 |
| O-Phosphorylethanolamine | Pearson Correlation | -0.121^*^ | 0.098 | 0.118^*^ |
|  | Sig. (2-tailed) | 0.038 | 0.094 | 0.043 |
|  | N | 291 | 295 | 295 |
| Saccharic acid -1 | Pearson Correlation | -0.101 | -0.147^*^ | -0.133^*^ |
|  | Sig. (2-tailed) | 0.086 | 0.012 | 0.022 |
|  | N | 291 | 295 | 295 |
| Glucosaminic acid -2 | Pearson Correlation | -0.094 | 0.141^*^ | 0.235^†^ |
|  | Sig. (2-tailed) | 0.110 | 0.015 | 0.000 |
|  | N | 291 | 295 | 295 |
| linoleic acid | Pearson Correlation | 0.007 | -0.126^*^ | -0.121^*^ |
|  | Sig. (2-tailed) | 0.902 | 0.030 | 0.037 |
|  | N | 291 | 295 | 295 |
| tryptophan 1 | Pearson Correlation | -0.096 | 0.093 | 0.271^†^ |
|  | Sig. (2-tailed) | 0.102 | 0.111 | 0.000 |
|  | N | 291 | 295 | 295 |
| Analyte 139 | Pearson Correlation | -0.089 | 0.078 | 0.207^†^ |
|  | Sig. (2-tailed) | 0.131 | 0.183 | 0.000 |
|  | N | 291 | 295 | 295 |
| Dioctyl phthalate | Pearson Correlation | -0.129^*^ | 0.091 | 0.235^†^ |
|  | Sig. (2-tailed) | 0.028 | 0.121 | 0.000 |
|  | N | 291 | 295 | 295 |
| Analyte 169 | Pearson Correlation | -0.151^*^ | 0.088 | 0.214^†^ |
|  | Sig. (2-tailed) | 0.010 | 0.133 | 0.000 |
|  | N | 291 | 295 | 295 |
| Triacontanoic acid, methyl ester -1 | Pearson Correlation | -0.107 | 0.095 | 0.188^†^ |
|  | Sig. (2-tailed) | 0.068 | 0.102 | 0.001 |
|  | N | 291 | 295 | 295 |
| Analyte 192 | Pearson Correlation | -0.119^*^ | 0.073 | 0.222^†^ |
|  | Sig. (2-tailed) | 0.042 | 0.214 | 0.000 |
|  | N | 291 | 295 | 295 |

^*^. Correlation is significant at the 0.05 level (2-tailed). ^†^. Correlation is significant at the 0.01 level (2-tailed). ^‡^. Ratios of parental relative metabolite levels were used in the analysis. YPP=Yield per plant, MSPH=Maturation stage plant height, HD=Heading date.


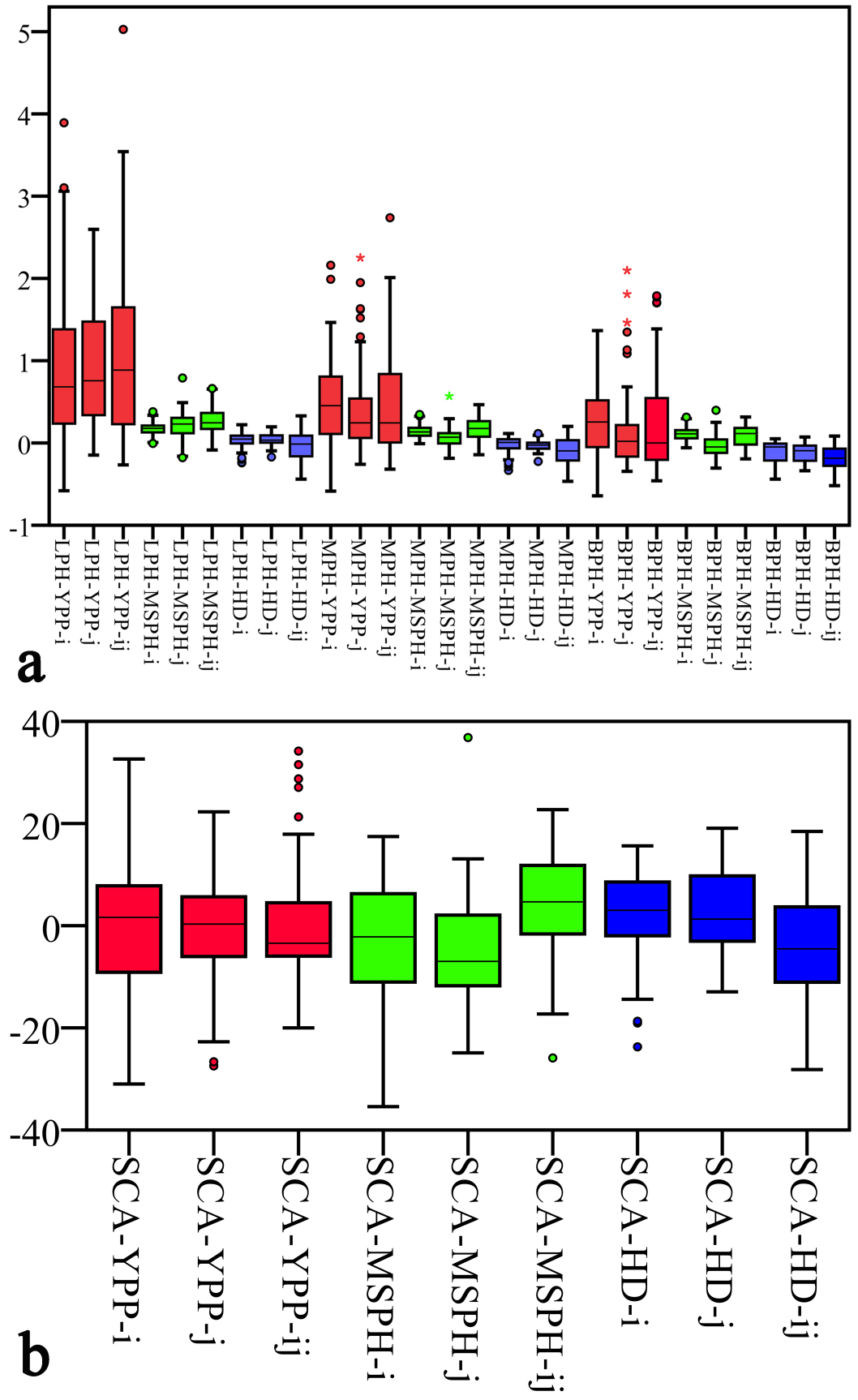


**Fig. S1. Hybrid performance of the three agronomic traits in i, j, and ij-group, respectively.** (**a**) Low-parent heterosis, mid-parent heterosis, and better-parent heterosis of the three traits were analyzed in i, j, and ij-group, respectively. (**b**) Specific combining ability of the three traits in i, j, and ij-group, respectively. YPP=Yield per plant, MSPH=Maturation stage plant height, HD=Heading date, LPH=Relative low-parent heterosis, MPH=Relative mid-parent heterosis, BPH=Relative better-parent heterosis, SCA=Specific combining ability.


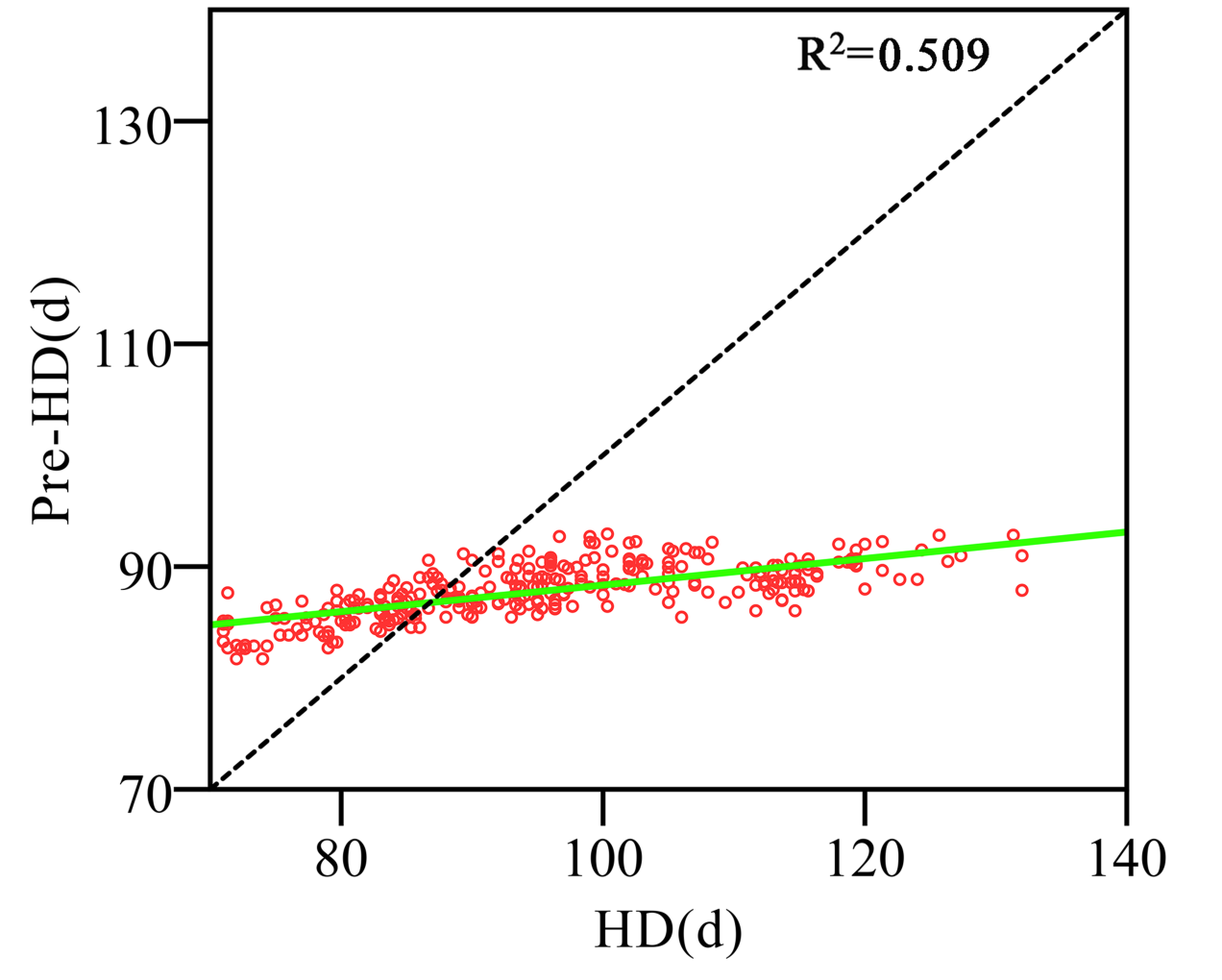


**Fig. S2. Prediction of hybrids’ heading date based on parental heading date.** The mean heading date values of the parents were used to predict hybrids’ heading date. Horizontal axis represents observed values and vertical axis represents predicted values. Green solid line is total fit line and black dotted line is *y*=*x*. HD=Heading date.
